# An entropic associative memory

**DOI:** 10.1038/s41598-021-86270-7

**Published:** 2021-03-25

**Authors:** Luis A. Pineda, Gibrán Fuentes, Rafael Morales

**Affiliations:** 1 Universidad Nacional Autónoma de México, IIMAS, 04510 Mexico City, Mexico; 2 Universidad de Guadalajara, SUV, 44130 Guadalajara, Mexico

**Keywords:** Psychology, Mathematics and computing

## Abstract

Natural memories are associative, declarative and distributed, and memory retrieval is a constructive operation. In addition, cues of objects that are not contained in the memory are rejected directly. Symbolic computing memories resemble natural memories in their declarative character, and information can be stored and recovered explicitly; however, they are reproductive rather than constructive, and lack the associative and distributed properties. Sub-symbolic memories developed within the connectionist or artificial neural networks paradigm are associative and distributed, but lack the declarative property, the capability of rejecting objects that are not included in the memory, and memory retrieval is also reproductive. In this paper we present a memory model that sustains the five properties of natural memories. We use Relational-Indeterminate Computing to model associative memory registers that hold distributed representations of individual objects. This mode of computing has an intrinsic computing entropy which measures the indeterminacy of representations. This parameter determines the operational characteristics of the memory. Associative registers are embedded in an architecture that maps concrete images expressed in modality specific buffers into abstract representations and vice versa. The framework has been used to model a visual memory holding the representations of hand-written digits. The system has been tested with a set of memory recognition and retrieval experiments with complete and severely occluded images. The results show that there is a range of entropy values, not too low and not too high, in which associative memory registers have a satisfactory performance. The experiments were implemented in a simulation using a standard computer with a GPU, but a parallel architecture may be built where the memory operations would take a very reduced number of computing steps.

## Associative memory

Natural memories of humans and other animals with a developed enough neural system are associative^1^. An image, a word or an odor can start a chain of remembrances on the basis of their meanings or contents. Information is accessed through cues, keys or descriptions, and memory retrieval is a constructive operation. The associative and constructive aspects of natural memories were systematically studied since F. Bartlett seminal work^2^ and associative memories have a central role in cognitive architectures, such as Kosslyn’s^3^. Natural memories contrast strongly with Random Access Memories (RAM) of standard digital computers in that the latter consists of place-holders—containing strings of symbols that are interpreted as representations—that are accessed through their addresses, and memory recovery is reproductive rather than constructive.

Computational models of associative memories have been extremely difficult to create within the symbolic paradigm, and although there have been important attempts using semantic networks since very early^4^ and production systems more recently^5^, practical symbolic associative memories are still lacking. This limitation was one of the original motivations for the parallel distributed processing program^6^, which questioned explicitly the capability of Turing Machines to properly address associative memories, among other high level cognitive functions—see the introduction of the cited Rumelhart’s book.

Associative memories have been studied within the artificial neural networks paradigm since Steinbuch’s Lernmatrix^7^, Willshaw’s correlograph^8^, Kohonen’s Linear Associator^9^ and the work of Gunter Palm on the capacity of associative memories^10^. A major breakthrough in this line of research was Hopfield’s model^11^ and later the Bidirectional Associative Memory^12^. Another contribution at the time was the neural networks and associative memories without weights^13,14^. However, these systems had a very limited performance. Further work by Ritter and collaborators on Morphological Associative Memory^15,16^ and Implicative Fuzzy Associative Memories^17,18^ provided the basis for practical applications. A more recent improvement is the extension of Hopfield’s model coupled with the use of convolutional neural networks that can be used in practical settings^19^.

Associative memories within the neural-networks paradigm store a large number of patterns that can be selected with complete or partial cues, but the memory does not generalize and the cue selects a pattern that has been input previously; in particular the pattern that is closest to the clue according to an abstract distant function. Hence, memory retrieval is reproductive but not constructive. A side effect of this conception of memory is that cues are never rejected and memory recall always renders an object. This limitation can be mitigated with heuristic strategies. For instance, if the network does not converge in *m* cycles for a given clue, it may be assumed that the pattern is not included; consequently realizing that something is not known takes a great deal of time, which is the opposite to common experience. Furthermore, the system can retrieve the wrong answer because it is not possible to know in advance that the query will converge due to the halting problem. This heuristic reflects analogous strategies used in symbolic representations where negation is equated with failure to proof, the so-called closed-world hypothesis, in opposition to the true or strong negation of natural language, logical languages, and knowledge-based systems supporting this kind of expressiveness^20,21^. These are significant differences in relation to natural memories where the distributed representation may generalize and hold patterns that have not been input explicitly; memory recovery is a constructive operation that renders a novel object; and memory reject is a sound, direct and efficient operation.

A more general limitation is that this kind of memories cannot hold symbolic or structured information, and associative memories in this paradigm are rather transfer functions mapping inputs into outputs for classification and prediction among other similar tasks, and the contention that such systems are not proper declarative memories still holds^22^.

In this paper we address such limitation and present an associative memory mechanism constituted by a number of associative memory registers that hold distributed representations of objects, and yet these can be registered, recognized and retrieved on the basis of a cue; the objects rendered by memory recall are novel constructions always; cues are rejected directly if the object is not in the memory; and the recovered objects can be expressed declaratively and interpreted as symbolic information.

## Relational-indeterminate computing

The present associative memory system is defined with a novel mode of computing that is referred to here as Relational-Indeterminate Computing (RIC)^23,24^. The basic object of computing in this mode is the mathematical relation, such that an object in the domain may be related to several objects in the codomain. The specification is presented by Pineda (2020)^23^—for a more general discussion see Pineda^25^—as follows:

Let the sets $A = \{a_1,\ldots ,a_n\}$ and $V = \{v_1,\ldots ,n_m\}$, of cardinalities *n* and *m*, be the domain and the codomain of a finite relation $r: A\rightarrow V$. The objects in the domain and codomain are referred to here as the *arguments* and the *values* respectively. For purposes of notation, for any relation *r* we define a function $R: A\times V\rightarrow \{0,1\}$—the relation in lower case and the function in upper case letters—such that $R(a_i,v_j) = 1$ or *true* if the argument $a_i$ is related to the value $v_j$ in *r*, and $R(a_i,v_j) = 0$ or *false* otherwise.

In this formalism, *evaluating a relation* is construed as selecting randomly one among the values associated to the given argument. In the same way that “$f(a_i) = v_j$” is interpreted as stating that the value of the function *f* for the argument $a_i$ is $v_j$, “$r(a_i) = v_j$” states that the value of the relation *r* for the argument $a_i$ is an object $v_j$ that is selected randomly -with an appropriate distribution—among the values for which $R(a_i,v_j)$ is true.

RIC has three basic operations: *abstraction*, *containment* and *reduction*. Let $r_f$ and $r_a$ be two arbitrary relations from *A* to *V*, and $f_a$ be a function with the same domain and codomain. The operations are defined as follows:

- Abstraction: $\lambda (r_f, r_a) = q$, such that $Q(a_i, v_j) = R_f(a_i, v_j) \vee R_a(a_i,v_j)$ for all $a_i \in A$ and $v_j \in V$ –i.e., $\lambda (r_f, r_a) = r_f \cup r_a$.
- Containment: $\eta (r_a, r_f)$ is true if $R_a(a_i,v_j) \rightarrow R_f(a_i,v_j)$ for all $a_i \in A$ and $v_j \in V$ (i.e., material implication), and false otherwise.
- Reduction: $\beta (f_a, r_f) = f_v$ such that, if $\eta (f_a,r_f)$ holds $f_v(a_i) = r_f(a_i)$ for all $a_i$, where the random distribution is centered around $f_a$, as elaborated below. If $\eta (f_a,r_f)$ does not hold, $\beta (f_a, r_f)$ is undefined—i.e., $f_v(a_i)$ is undefined—for all $a_i$.

Abstraction is a construction operation that produces the union of two relations. A function is a relation and can be an input to the abstraction operation. Any relation can be constructed out of the incremental abstraction of an appropriate set of functions. The construction can be pictured graphically by overlapping the graphical representation of the included functions on an empty table, such that the columns correspond to the arguments, the rows to the values and the functional relation is depicted by a mark in the intersecting cells.

The containment operation verifies whether all the values associated to an argument $a_i$ in $r_a$ are associated to the same argument in $r_f$ for all the arguments, such that $r_a \subseteq r_f$. The containment relation is false only in case $R_a(a_i,v_j)=1$ and $R_f(a_i,v_j)=0$—or if $R_a(a_i,v_j) > R_f(a_i,v_j)$—for at least one $(a_i,v_j)$.

The set of functions that are contained in a relation, which is referred to here as the *constituent functions*, may be larger than the set used in its construction. The constituent functions are the combinations that can be formed by taking one value among the ones that the relation assigns to an argument, for all the arguments. The table format allows to perform the abstraction operation by direct manipulation and the containment test by inspection. The construction consists on forming a function by taking a value corresponding to a marked cell of each column, for all values and for all columns. The containment is carried on by verifying whether the table representing the function is contained within the table representing the relation by testing all the corresponding cells through material implication.

For this, the abstraction operation and the containment test are productive. This is analogous to the generalization power of standard supervised machine learning algorithms that recognize not only the objects included in the training set but also other objects that are similar enough to the objects in such set.

Reduction is the functional application operation. If the argument function $f_a$ is contained in the relation $r_f$, reduction generates a new function such that its value for each of its arguments is selected from the values in the relation $r_f$ for the same argument. In the basic case, the selection function is the identity function—i.e., $\beta (f_a, r_f) = f_a$. However, $\beta$ is a constructive operation such that the argument function $f_a$ is the cue for another function recovered from $r_f$, such that $v_j$ is selected from $\{v_j| (a_i, v_j)\in r_f\}$ using and appropriate random distribution function centered around $f_a(a_i)$. If $f_a$ is not contained in $r_f$ the value of such functional application operation is not defined.

Relations have an associated entropy, which is defined here as the average indeterminacy of the relation *r*. Let $\mu _i$ be the number of values assigned to the argument $a_i$ in *r*; let $\nu _i$ = $1/\mu _i$ and *n* the number of arguments in the domain. In case *r* is partial, we define $\nu _i = 1$ for all $a_i$ not included in *r*. The *computational entropy* *e*(*r*)—or the entropy of a relation—is defined here as:

$$\begin{aligned} e(r) = -\frac{1}{n} \sum _{i=1}^{n} \log _2(\nu _i). \end{aligned}$$

A function is a relation that has at most one value for any of its arguments, and its entropy is zero. Partial functions do not have a value for all the arguments, but this is fully determined and the entropy of partial functions is also zero.

## Table computing

The implementation of RIC in table format is referred to as *Table Computing*^24^ . The representation consists of a set of tables with *n* columns and *m* rows, where each table is an *Associative Register* that contains a relation between a set of arguments $A = \{a_1,\ldots , a_n\}$ and a set of values $V = \{v_1,\ldots ,v_m\}$. Let $[R_k]^t$ be the content of the register $R_k$ at time *t* and $\leftarrow$ an assignment operator such that $R_k \leftarrow R_j$ assigns $[R_j]^t$ to $[R_k]^{t+1}$, where *j* and *k* may be equal. This corresponds to the standard assignment operator of imperative programming languages. The machine also includes the conditional operator *if* relating a condition *pred* to the operations $op_1$ and $op_2$—i.e., if *pred* then $op_1$ else $op_2$, where $op_2$ is optional. The initialization of a register *R* such that all its cells are set to 0 or to 1 is denoted $R\leftarrow 0$ or $R\leftarrow 1$ respectively, and $R\leftarrow f$ denotes that a function $f:A\rightarrow V$ is input into the register *R*. The system also includes the operators $\lambda$, $\eta$ and $\beta$ for computing the corresponding operations. These are all the operations in table computing.

Let *K* be a class, $O_k$ a set of objects of class *K*, and $F_O$ a set of functions with *n* arguments and *m* values, such that each function $f_i \in F_O$ represents a concrete instance $o_i \in O_k$ in terms of *n* features, each associated to one of *m* possible discrete values. The function $f_i$ may be partial—i.e., some features may have no value.

Let $R_k$ be an associative memory register and $R_{k-{i/o}}$ an auxiliary input and output register, both of size $n \times m$. The distributed representation of the objects $O_k$ is created by the algorithm $\textit{Memory\_Register}(f_i, R_k)$ for all $f_i \in F_O$ as follows:

- $$\textit{Memory\_Register}(f_i, R_k)$$:
  1. $$R_{k-{i/o}}\leftarrow f_i$$
  2. $$R_k \leftarrow \lambda (R_k, R_{k-{i/o}})$$
  3. $$R_{k-{i/o}}\leftarrow 0$$

The recognition of an object $o \in O_k$—or of class *K*– represented by the function *f*, is performed by the algorithm *Memory_Recognize* as follows:

- $$\textit{Memory\_Recognize}(f, R_k)$$:
  1. $$R_{k-{i/o}}\leftarrow f$$
  2. If $$\eta (R_{k-{i/o}},R_k)$$ then $$(R_{k-{i/o}}\leftarrow 1)$$

The retrieve or recovery of an object is performed through the reduction operation. This is a constructive operation that recovers the content of the memory in the basis of the cue. The procedure is as follows:

- $$\textit{Memory\_Retrieve}(f, R_k)$$:
  1. $$R_{k-{i/o}}\leftarrow f$$
  2. If $\eta (R_{k-{i/o}},R_k)$ then $R_{k-{i/o}}\leftarrow \beta (R_{k-{i/o}},R_k)$ else $R_{k-{i/o}}\leftarrow 0$

The standard input configuration of this machine specifies that the auxiliary register contains the function to be registered or recognized, or the cue of a retrieve, at the initial state of the corresponding memory operation. This condition is enforced at step (1) of the three memory procedures. The standard output specifies the content of the auxiliary register when the computation is ended. It is 0 when the memory register operation is ended and when the cue is rejected in a retrieve operation, and 1 if recognition is successful; otherwise the object to be recognized is rejected and if the retrieve operation is successful it contains the function recovered out of the cue.

The interpretation conventions state that the content of the associative register is interpreted as an abstract concept, and the content of the input auxiliary register is interpreted as a concrete concept—the concept of an individual object.

## Architecture

**Figure 1:**
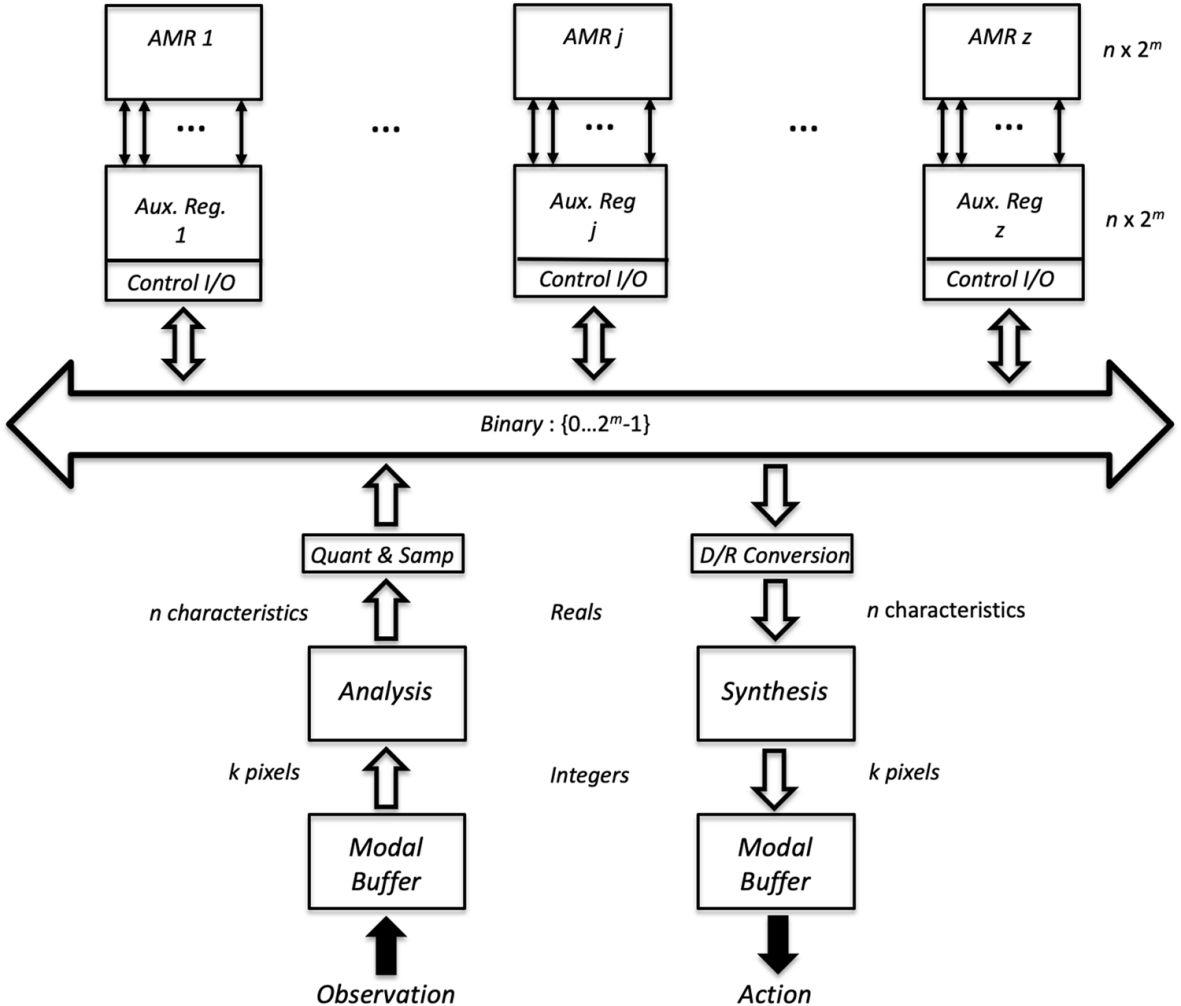
Associative memory architecture.

Table Computing was used to implement a novel associative memory system. The conceptual architecture is illustrated in Fig. 1. The main components are:

- A set of *Associative Memory Registers* (AMRs) of size $n \times 2^m$ for $n \ge 1$ and $m \ge 0$ with their corresponding *Auxiliary Registers* (Aux. Reg.) each having a *Control I/O* unit.
- A central control unit sending the operation to be performed to all AMRs, and receiving the final status of the operation (i.e., whether it was successful or not) and the entropy of each AMR (not shown in the diagram).
- A bus with *n* tracks, each representing a characteristic or feature, with its corresponding value: a binary number from 0 to $2^m-1$ at a particular time.
- An input processing unit constituted by:
  - An input modal pixel buffer with the concrete representation of images produced by the observations made by the computing agent directly. For instance, the input buffer can contain *k* pixels with 256 gray levels represented by integers from 0 to 255.
  - An analysis module mapping concrete representations to abstract modality-independent representations constituted by *n* characteristics with their corresponding real values.
  - c) A quantizing and sampling module mapping the real values of the *n* characteristics into $2^m$ levels, represented by binary digits of length *m*, which are written on the corresponding track of the bus.
- An output processing unit constituted by:
  - A Digital/Real conversion module that maps binary numbers in a track of the bus to real numbers, for the *n* tracks.
  - A synthesis module mapping abstract modality-independent representations constituted by *n* characteristics with their corresponding values into concrete representations with *k* pixels with their values.
  - An output modal buffer with the concrete representation of images produced by the synthesis module. The contents of the output buffer are rendered by an appropriate device, and constitute the *actions* of the system.

The bus holds the representations of functions with domain $A = \{a_1,\ldots ,a_n\}$ and range $V = \{v_1,\ldots ,v_n\}$ where $0\le v_j \le 2^m-1$ that represent the objects that are stored, recognized or recovered from the AMRs by the corresponding operations. Objects are placed on the bus through an input protocol; and recovered objects from the AMR are rendered through an output protocol, as follows:

- *Input protocol*:
  1. Sense the object $o_i$ and place it in the input modal buffer;
  2. Produce its abstract representation $f_i$ through the analysis module;
  3. Produce its corresponding quantized representation and write it on the bus;
  4. Write the value of all *n* arguments diagrammatically into the corresponding row of the auxiliary register $R_{k-{i/o}}$;
- *Output Protocol*:
  1. Write the content of the auxiliary register $R_{k-{i/o}}$ on the bus as binary numbers with *m* digits for all *n* arguments;
  2. Transform the digital values of the *n* characteristics into real values;
  3. Generate the concrete representation of the object and place it on the output buffer through the synthesis module.

The core operations of the memory register, memory recognition and memory retrieve algorithms are carried on directly on the AMRs and their corresponding auxiliary registers in two or three computing steps—i.e., the operations $\lambda$, $\eta$ and $\beta$ in addition to the corresponding assignment operations. The assignments $R_{k-{i/o}}\leftarrow f_i$, $R_{k-{i/o}}\leftarrow 0$ and $R_{k-{i/o}}\leftarrow 1$ are carried out in a single computing step.

The memory operations use the input and output protocols, and are performed as follows:

- $Memory\_Register(f_i, R_k)$: The register $AMR_k$ is set on, and the remaining AMRs are set off; the input protocol is performed; the $Memory\_Register$ operation is performed.
- $Memory\_Recognize(f_i, R_k)$: All AMRs are set on; the input protocol is performed; the $Memory\_Recognize$ operation is performed; all AMRs send its status and entropy to the control unit; if no AMR’s recognition operation is successful the object is rejected.
- $Memory\_Retrieve(f_i, R_k)$: All AMRs are set on; the input protocol is performed; the $Memory\_Retrieve$ operation is performed; all AMRs send its status and entropy to the control unit; all AMRs but the selected one are set off; the output protocol is executed; the recovered object is placed on the output buffer.

## Analysis and synthesis

The concrete information that is sensed from the environment and placed in the input buffer—where the characteristics stand for external signals—is mapped by the Analysis Module into the abstract representation that characterizes the membership of the object within a class. Both concrete and abstract representations are expressed as functions, but while in the former case the arguments have a spatial interpretation—such as the pixels of an image—in the latter the functions stand for modality-independent information.

**Figure 2:**
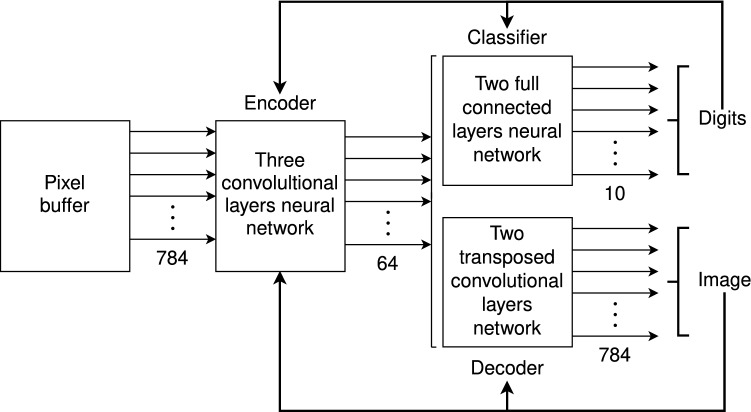
Training the analysis and synthesis modules.

The analysis module in the present architecture is implemented with a neural network with three convolutional layers, which is called *encoder*^26^, as illustrated in Fig. 2. The synthesis module is implemented as a transposed convolutional neural network with two layers, which is called *decoder*, mapping sets of features into their corresponding images. The encoder and the decoder conform what is called an *autencoder*^27,28^. The training phase was configured by adding a classifier, which is a fully connected neural network (FCNN) with two layers, mapping sets of features output by the encoder into their corresponding digits.

The diagram shows the case in which the encoder has 784 input features, corresponding to a pixel buffer of size $28 \times 28$, each taking one out of 256 gray levels, while its output is a function with 64 arguments with real values. The encoder, the decoder and the classifier were trained together, in a supervised manner by standard back-propagation.

Once the autoencoder has been trained the classifier is removed; the encoder is used as the analysis module; and the decoder is used as the synthesis module, mapping the information retrieved from the AMRs into the output buffer.

## A visual memory for hand written digits

The associative memory system was simulated through the construction of a visual memory for storing and retrieving distributed representations of hand written digits from “0” to “9”. The system was built and tested using the MNIST database available at http://yann.lecun.com/exdb/mnist/. In this resource each digit is defined as a 28 $\times$ 28 pixel array with 256 gray levels. There are 70,000 instances available. The instances of the ten digit types are mostly balanced.

The MNIST corpus is a widely available and well-known resource for learning and practising with convolutional neural networks (CNN), and very high recognition results are achieved with current machine learning technology. MNIST has also been used to model classification with noise^29^, but CNN do not perform well when the digits are occluded although the task can be improved combining CNN with compositional models^30^. The digits in this data-set can be reproduced with high accuracy with the so-called variational autoencoders and generative adversarial networks. However, generative techniques in the neural networks paradigm always produce an object despite that the amount of information in the input may be too low; for instance, the occlusion may be too severe and people may be unable to tell whether there is a digit, which one is it or what is its shape, but the synthesis network will nevertheless render an object. An associative memory with the capability of rejecting a cue—identifying true negatives—may prevent such a kind of miss-generation, as illustrated below. This is an open research problem that, as far as we are aware of, has not been addressed in generative networks and, in particular, using the MNIST corpus.

MNIST has also been used to asses the classification performance of an associative memory in relation to standard deep neural networks^31^ and to model biological associative memories^32^ but the inclusion of an associative memory in a cognitive architecture in which the images are interpreted and generated not only on the basis of bottom-up processes but also with the top-down information stored in an associative memory has not been investigated with this dataset, hence the recognition and retrieval performance of highly occluded objects preventing the generation of false positives in experiment 5 below are novel. The constructive nature of memory retrieval as opposed to the reproductive character of neural network models is also a novel use of this set. For these reasons MNIST can be seen as a current baseline resource to asses the properties and performance of this kind of systems.

The corpus was partitioned further in three disjoint sets:

- Training Corpus (*TrainCorpus*): For training the analysis and synthesis modules (57 %).
- Remembered Corpus (*RemCorpus*): For filling in the Associative Memory Registers (33 %).
- Test Corpus (*TestCorpus*): For testing (10 %).

The corpus partitions were rotated through a standard 10-fold cross-validation procedure. Five experiments supported by a given analysis and synthesis modules were performed:

1. Experiment 1: Define an associative memory system including an AMR for holding the distributed representation of each one of the ten digits. Determine the precision and recall of the individual AMRs and of the overall system. Identify the size of the AMRs with satisfactory performance.
2. Experiment 2: Investigate whether AMRs can hold distributed representations of objects of different classes. For this an associative memory system including an AMR for holding the distributed representation of two “overlapped” digits is defined. Determine the precision and recall of the individual AMRs and of the overall system.
3. Experiment 3: Determine the overall precision and recall for different levels of entropy of the AMRs, for the AMR size with the best performance, as identified in Experiment 1.
4. Experiment 4: Retrieve objects out of a cue for different levels of entropy and generate their corresponding images—with the same AMRs used in experiment 3. Assess the similarity between the cue and the recovered object at different levels of entropy.
5. Experiment 5: Retrieve digits of significantly occluded objects. Assess the precision and recall and the quality of the generated images.

In all five experiments each instance digit is mapped into a set of 64 features through the analysis module. Hence, each instance is represented as a function $f_i$ with domain $\{a_1,\ldots ,a_{64}\}$ where each argument $a_i$ is mapped to a real value $v_i$—i.e. $f_i(a_i) = v_j$. The values are quantized in $2^m$ levels. The tables or associative registers have sizes of $64 \times 2^m$. The parameter *m* determines the granularity of the table. The experiments one and two were performed with granularities $2^m$ for $0<=m<=9$. So, the memory was tested with 10 granularities in each setting. The source code for replicating the experiments, including the detailed results and the specifications of the hardware used, are available in Github at https://github.com/LA-Pineda/Associative-Memory-Experiments.

### Experiment 1

Compute the characteristics of AMR of size $64 \times 2^m$ for $0\le m \le 9$:

1. Register the totality of *RemCorpus* in their corresponding register through the $Memory\_Register$ operation;
2. Test the recognition performance of all the instances of the test corpus through the $Memory\_Recognize$ operation;
3. Compute the average precision, recall and entropy of individual memories.
4. Select a unique object to be recovered by the $Memory\_Retrieve$ operation; compute the average precision and recall of the integrated system when this choice has been made.

**Figure 3:**
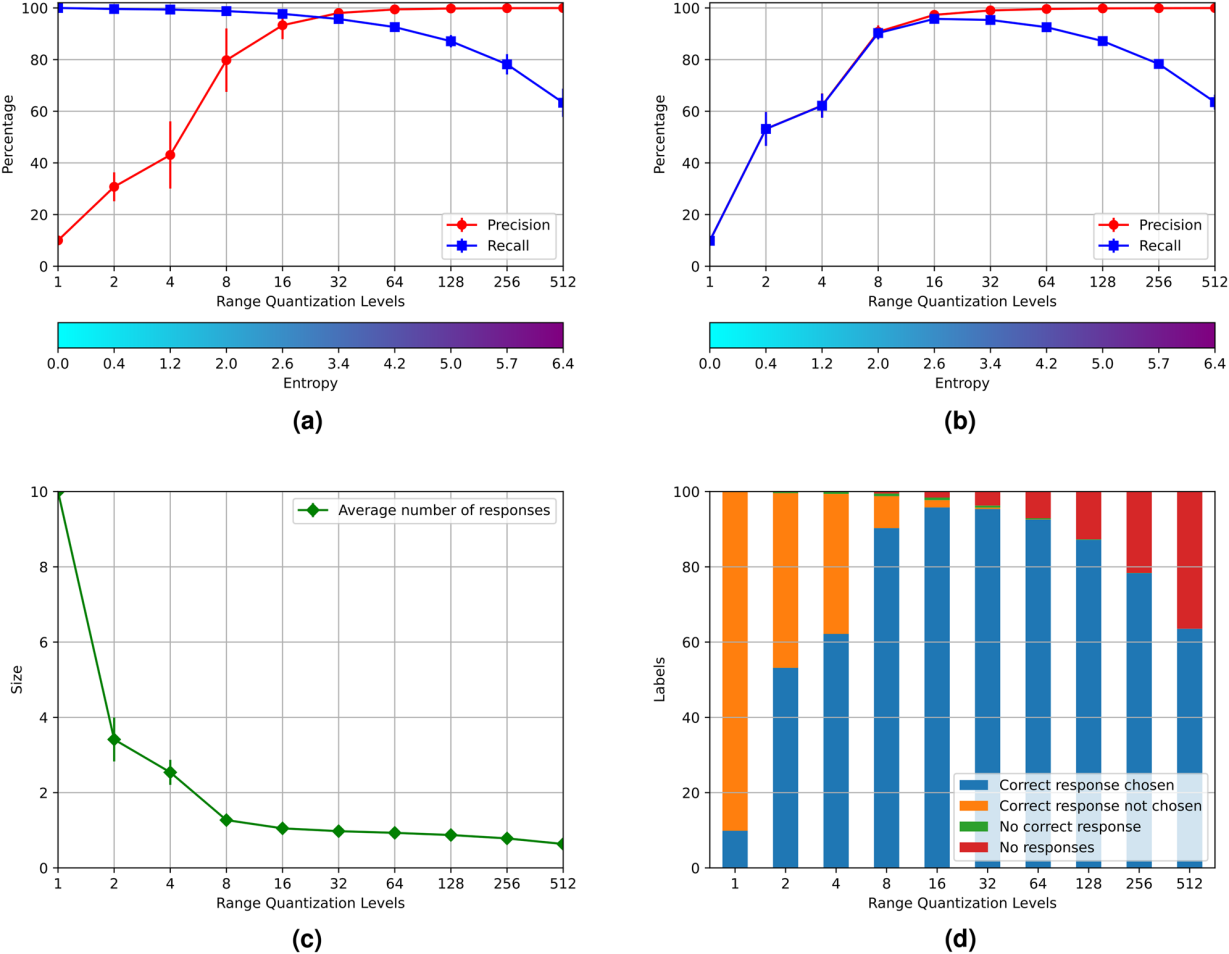
Results of experiment 1.

The average precision, recall and entropy of the ten AMRs are shown in Fig. 3a. The precision for the smallest AMR with only one row are $10 \%$—the proportion of the test data of each digit—and recall is $100 \%$—as all the information is confused and everything is accepted. The precision grows with the size of the AMRs and has a very satisfactory value up from 32 rows. The recall, on its part, remains high until the granularity of the table is too fine and it starts to decrease. The entropy is increased almost linearly with the AMRs size, starting from 0 where the relations have only one value.

The average precision, recall and entropy of the integrated system is shown in Fig. 3b. The precision has a similar pattern to the one above, but the recall lowers significantly in AMRs with a small *m*—the precision and recall are the same practically for $m \le 4$. The reason of this decrease is that when the size of the AMR is small, there is a large number of false positives, and several AMRs different from the right one may accept the object; however, one register must be selected for the memory retrieve operation, and there is no information to decide which one. This decision was made using the AMR with the minimal entropy, although this choice does not improve over a random choice using a uniform distribution.

The average number of accepting AMRs for each instance per AMR size is shown in Fig. 3c. As can be seen this number goes from 10 for AMRs with one row to 1 for AMRs with 8 and 16 rows, where the precision is very high because every AMR recognizes only one instance in average. This effect is further illustrated in Fig. 3d.

### Experiment 2

**Figure 4:**
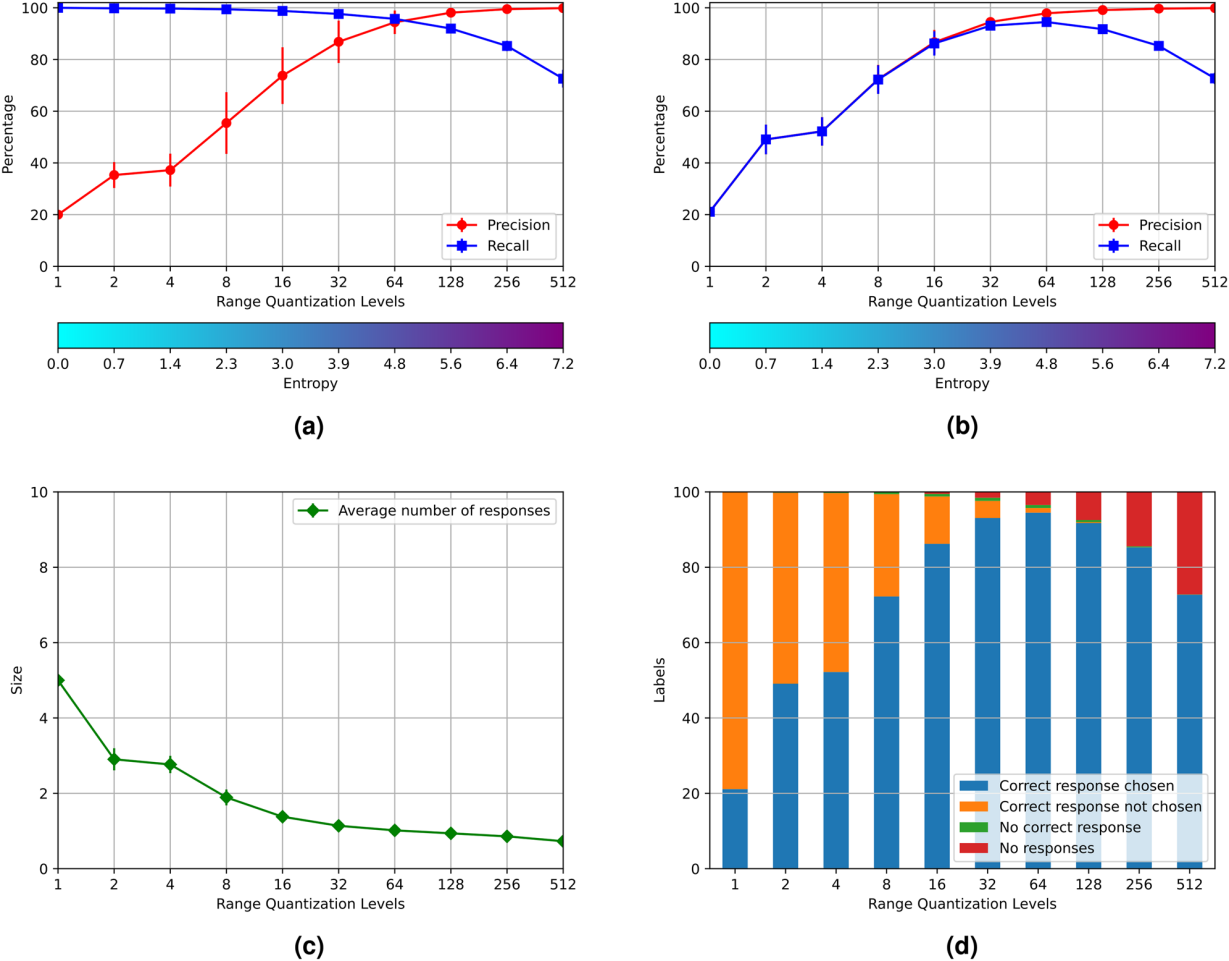
Results of experiment 2.

In this experiment each associative register holds the representation of two different digits: “0” and “1”, “2” and “3”, “4” and “5”, “6” and “7” and “8” and “9”. The procedure is analogous to experiment 1. The results of the experiment are shown in Fig. 4. The results of both experiments are also analogous, with the only difference that the entropy of the AMRs holding two digits are larger than the entropies of the AMRs holding a single digit. This experiment shows that it is possible to create associative memories holding overlapped distributed representations of more than one individual object, that have a satisfactory performance.

### Experiment 3

The purpose of this experiment was to investigate the performance of an AMR with satisfactory operational characteristics in relation to its entropy or information content. Experiment 1 shows that AMRs with sizes $64 \times 32$ and $64 \times 64$ satisfy this requirement. As their performance are practically the same, the smallest one was chosen for a basic economy criteria.

**Figure 5:**
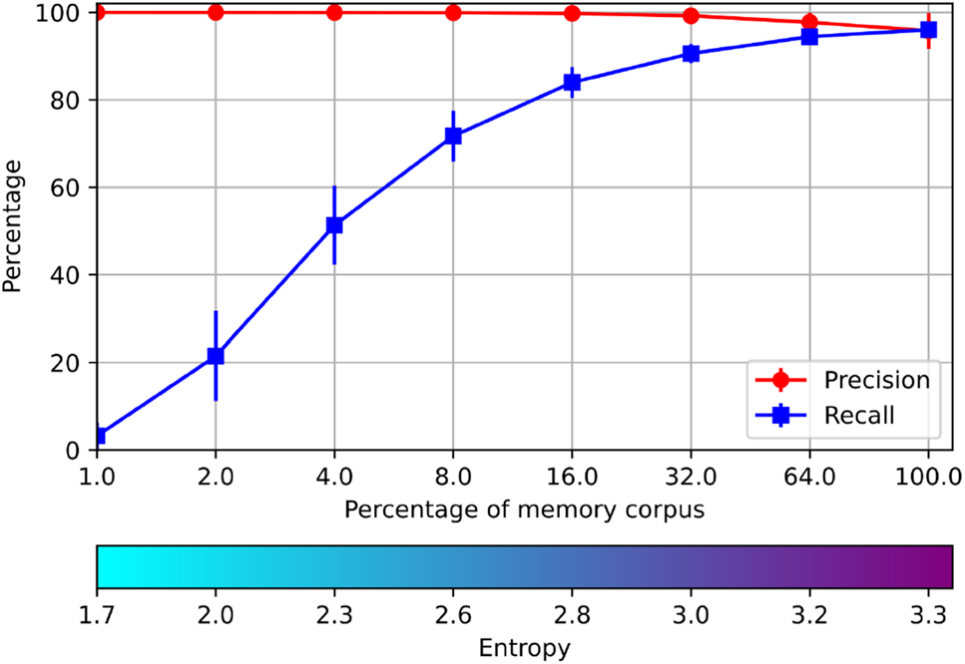
Results of experiment 3.

The AMRs were filled up with varying proportions of the *RemCorpus*—1 %, 2 %, 4 %, 8 %, 16 %, 32 %, 64 % and 100 %, as shown in Fig. 5. The entropy increases according to the amount of remembered data, as expected. Precision is very high for very low entropy values and it decreases slightly when the entropy is increased, although it remains very high when the whole of *RemCorpus* is considered. Recall, on its part, is very low for very low levels of entropy but increases very rapidly when the AMR is filled up with more data.

### Experiment 4

This experiment consists on assessing the similarity of the objects retrieved from the memory with the cue. As memory retrieve is a constructive operation that renders a novel object, the recovered object may be somewhat different from the cue. This is constructed by the $\beta$ operation, as described above. In the present experiment a random triangular distribution is used for selecting the values of the arguments of the retrieved object out of the potential values of the AMR for the corresponding arguments.

The hypothesis is that the increase of memory recall goes in hand with higher entropy, but the space of indeterminacy of the AMR impacts negatively in the resemblance or similarity between the cue and the retrieved object.

**Figure 6:**
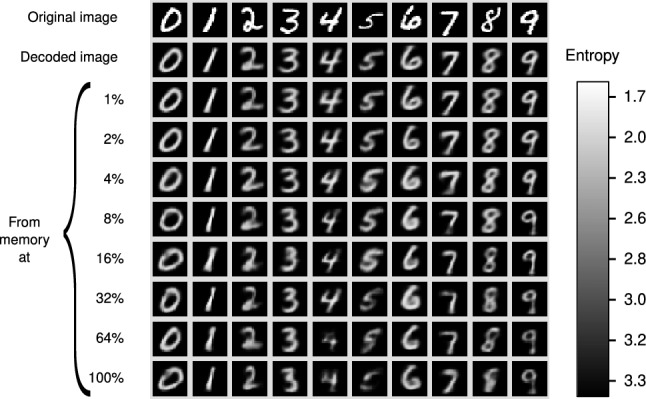
Similarity between the cue and the recovered digits as a function of the entropy.

Figure 6 illustrates an instance of each of the ten digits at different levels of entropy, where each column shows a digit retrieved and reconstructed with the same cue. All the examples in this Figure are accepted by the recognition operation at all levels of entropy and the intention is to illustrate the quality of reconstruction when the entropy is increased.

The first row contains the cue for the retrieval operation; the second is the decoded image, which corresponds to the one resulting from directly synthesizing the output of the analysis module. This is equivalent to specify $\beta$ as the identity function—although such choice would remove the constructive aspect of the memory retrieve operation, and memory recognition and memory retrieve would amount to the same operation. The decoded image is very similar to the cue, but it is not an exact copy because the autoencoder produces an approximation.

The remaining images, from top to bottom, correspond to the retrieved objects for the nine levels of the *RemCorpus* that are considered (the codified image corresponds to $e = 0$). The AMRs are used within their operational range and the quality of the digits remains mostly high at different levels, although it starts to decrease when the entropy reaches a value of around 3.0 for some of the digits.

**Figure 7:**
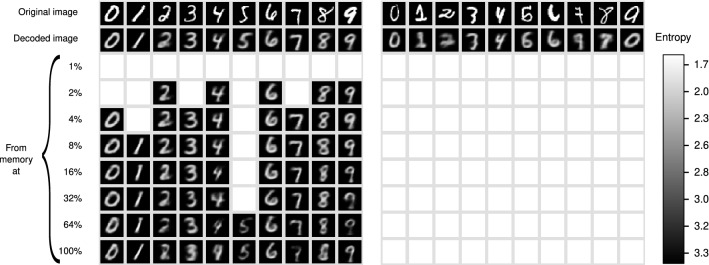
Memory recovery with rejection.

Figure 7 illustrates an instance of each of the ten digits which are rejected at some entropy levels, and their image is not generated at such levels. This is illustrated with blank squares. The left figure shows instances in which the digits are rejected at low levels of entropy, but they are accepted at higher levels, in which case its image is constructed. Although the quality of the digits is satisfactory at moderate levels of entropy, it decreases at higher levels.

The right figure shows examples in which the digits are rejected at all entropy levels. These instances are extreme cases of their classes, and may be confused even by human interpreters if shown without the context. For instance, the “8” resembles the Greek letter $\gamma$, and the ‘9” could be taken by an *a*. However, the autoencoder does generate symbols in these examples, the 7 and the 0 respectively, although this is clearly wrong. This is an important limitation of classifiers that have no clear notion of rejection and always approximate to the most similar object according to an abstract and implicit similarity measure. In addition, the shape of the figure is mostly a prototypical object produced by the network and there is no natural way to assess whether it does resemble the original object. Furthermore, the object generated may be a false positive of the rendered class.

### Experiment 5

**Figure 8:**
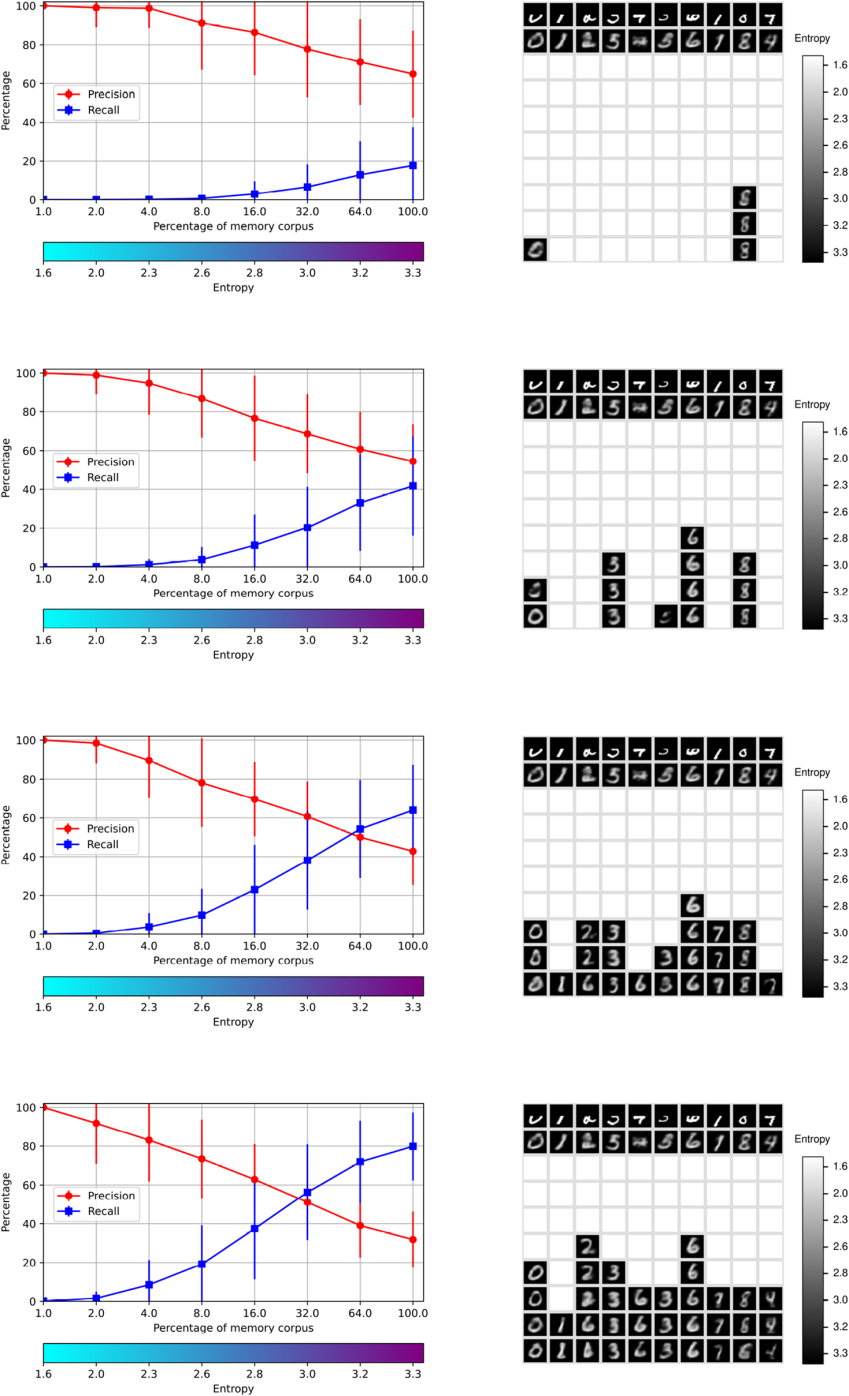
Memory retrieval with occlusions of $50\%$ at the top with relaxations of 1, 2 and 3 features out of 64.

**Figure 9:**
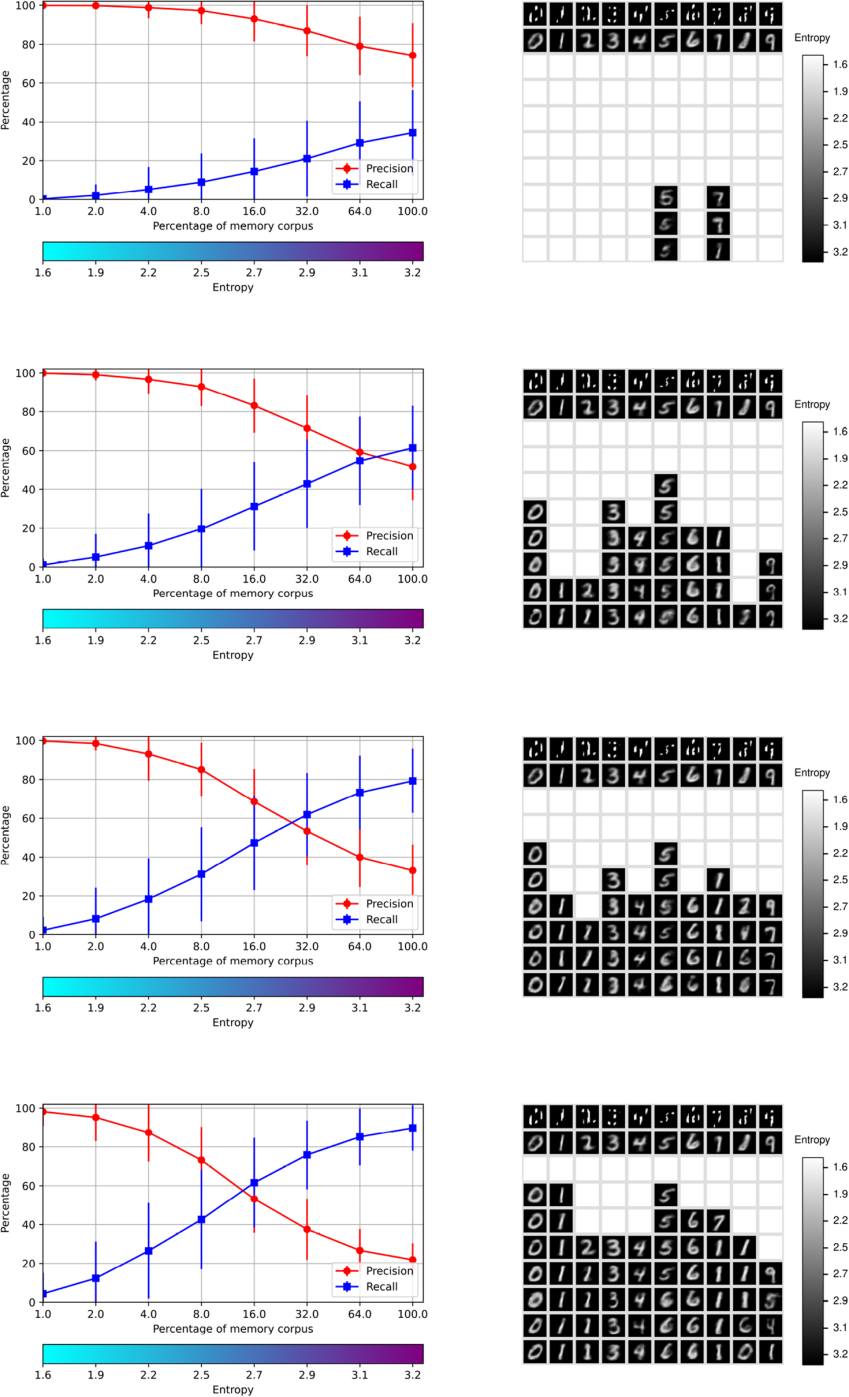
Memory retrieval with occlusions with vertical bars with relaxations of 1, 2 and 3 features.

The purpose of this experiment is to illustrate the performance of the memory system in the recognition, retrieval and rendering operations of significantly occluded digits. The recovery of patterns with incomplete information, such as noise and occlusions, has been one important motivation for the study and development of associative memories since the original proposals. This concern is addressed next. For this experiment the *RemCorpus* was used to fill the AMRs and the autoencoder was trained with the *TrainCorpus* as before. The digits in the *TestCorpus* were occluded by an opaque rectangle at the top $50\%$ of the input buffer, as shown in the the pictures at the right in Fig. 8, and by vertical bars of four pixels wide in the input buffer, as shown in Fig. 9. The graphs at the left show the corresponding precision, recall and entropy levels. The blank squares denote that the digits are not accepted, and hence their images not generated at the corresponding entropy level, as before.

The expectation is that the incomplete information in the input increases the ambiguity of the pictures and, consequently, that the cues are accepted more easily—with the corresponding increase of recall—and that the precision is decreased according to the size of the occlusion; however, if the amount of information is too low, the cues should be rejected directly. This effect is appreciated in the graph and pictures at the top of Figs. 8 and 9. However, the $Memory\_Recognize$ operation is too strict, as a digit is accepted only if all $100\%$ of abstract features characterizing it are accepted too, and it only requires one feature to fail for the digit to be rejected. For this, recall is too low for significantly incomplete cues at low levels of entropy.

To allow for a more flexible behavior, the recognition operation can be relaxed and the cue can be accepted if a given percentage of features that fail the test is allowed. The number of features in the current experimental setting is 64, and effects of the relaxation of 1, 2 and 3 features—$1.6\%$, $3.1\%$ and $4.7\%$—are shown respectively on the second, third and four levels of Figs. 8 and 9. Relaxing the recognition test impacts on a decrease in precision, as can also be seen in the figures, and the memory retrieval operation produces alternative hypothesis. This effect is better appreciated in the bottom row of Fig. 8 in which three features are relaxed, where 2 is interpreted as 2, 6 and perhaps 4; 4 as 6 and 4; and 8 as 8 and 6, although the quality of the images is very poor at higher levels of entropy. It is possible that all the hypotheses are wrong, but nevertheless these may be useful if they are interpreted further in relation to a wider context. More generally, there is a trade-off between precision and recall when the input digits are occluded significantly.

In this setting the autoencoder always generates a picture, as in the case with complete information, despite that the occlusion may be significant. This emphasizes that the networks that are unable to reject always generate an object, but its shape is depends more on the weights of the network than on the actual cue.

## Experimental setting

The programming for all the experiments was carried out in Python 3.8 on the Anaconda distribution. The neural networks were implemented with TensorFlow 2.3.0, and most of the graphs produced using Matplotlib. The experiments were run on an Alienware Aurora R5 with an Intel Core i7-6700 Processor, 16 GBytes of RAM and an NVIDIA GeForce GTX 1080 graphics card. The images shown together in Fig. 6 were selected one column per experimental run, and the criteria for selection was to have recognition of the digits in the first (1%) and last (100%) stage. The digits for Fig. 7a where chosen randomly, one per experimental run, whereas the digits for Fig. 7b where chosen such that they were not recognized in the last stage (100%).

Regarding the neural networks, firstly the image classifier (encoder-classifer) and the autoencoder (encoder-decoder) were trained together using MNIST data—in the first version of the experiments, they were trained separately, but the results were not as good. Secondly, the features for all images in MNIST were generated and stored using only the trained encoder, and experiments 1 to 3 where carried out. Thirdly, the decoder was used to generate the images for Experiment 4 from the features produced by the memory retrieve algorithm in Experiments 3. Finally, the encoder was used to produce the features for the occluded images from the *TestCorpus*, the process of Experiment 3 was repeated several times using different degrees of tolerance, and the decoder was used to produce the new images, as shown in Fig. 7.

## Discussion

In this paper we presented a memory system that is associative and distributed but declarative, in which memory retrieval is a constructive operation and cues of objects not included in the memory are rejected directly. Individual instances of represented objects are characterized at three different levels: (i) as concrete modality-specific representations in the input and output buffers –which can be sensed or rendered directly—or, alternatively, as functions from pixels to values; (ii) as abstract modality-independent representations in a space of characteristics, which are functions standing in a one-to-one relation to their corresponding concrete representations in the first level; and (iii) as distributed representations holding the disjunctive abstraction of a number, possibly large, of instance objects expressed in the second level. The first level can be considered as declarative and symbolic; the second is still declarative but is independent of representation, so can hold and integrate representations of objects presented in different modalities; and the third is a sub-symbolic structure holding the abstraction of a set of objects of the second level.

The memory register and recognition operations use only logical disjunction and material implication, which are performed by direct manipulation, cell to cell in the tables, and information is taken and placed on the bus by direct manipulation too, enhancing the declarative aspect of the system. The associative property depends on the dual role played by the intermediate representations that express content and, at the same time, select their corresponding Associative Memory Registers through the memory recognition and retrieval operations. The memory register operation is analogous to the training phase of supervised machine learning, and it presupposes an attention mechanism that selects the AMR in which the information is input. Addressing this restriction is left for further work.

The analysis and synthesis modules, mapping concrete into abstract representations and vice versa, are serial computations from a conceptual perspective—although their internal micro-operations can be performed in parallel using GPUs—but the memory operations manipulate the symbols stored in the corresponding cells of the tables directly, taking very few computing steps, which can be performed in parallel if the appropriate hardware is made available. In the present architecture the memory operations involve the simultaneous activation of all the associative memory registers, and this parallelism takes places not only at the algorithmic and implementation levels but also at the computational or functional system level, in Marr’s sense^33^. The analysis and synthesis mechanisms are implemented here through standard deep neural networks, but this is a contingency. From the conceptual perspective this functionality can be achieved with other modes of computing, that map concrete representations into the abstract characteristics space by other means.

The functionality of the memory proper can be also distinguished from the input and output processes in terms of the indeterminacy of the computing objects. The analysis and synthesis modules compute a function whose domain and range are sets of functions, and these processes are fully determined: provide the same value for the same argument always. Hence, these are zero entropy computations. However, the distributed representations stored in the memory registers have a degree of indeterminacy, which is measured by the computing entropy. The entropy is a parameter of the system performance, as can be seen in the five experiments. First, it measures the operational range of the associative registers, as shown in experiments 1 and 2. If the entropy is too low, precision and recall are low overall, but if it is too high, recall is also diminished. However, there is an entropy level in which both precision and recall are pretty satisfactory. The experiments showed that memory registers with sizes of $64 \times 32$ and $64 \times 64$ have satisfactory operational characteristics. The smaller register was chosen for the further experiments due to basic economy considerations.

Experiment 2 shows that a single associative memory register can hold the distributed representation of more than one object. The cost is that the entropy is increased, and larger registers with a large amount of information are required for the construction of operational memories. However, this functionality is essential, both for the construction of higher abstractions, and possibly for the definition of composite concepts. This is also left for further work. The point to stress here is that the measure involved is again the entropy.

Experiment 3 addressed the question of what is the amount of information and the level of entropy that is required for effective memory recognition and retrieval, given an operational memory register. The results show that recognition precision is very high regardless the amount of information that is feed into the memory register. Hence, whenever something is accepted one can be pretty sure that it belongs to the corresponding class. However, recognition recall is very low for low levels of entropy but becomes very high even with a moderate amount of information. However, if the information is increased with noise, the entropy has a very large value, and although recognition recall will not decrease, the information is confused and recognition precision will lower significantly. Hence, there is again a range of entropy, not too low and not too high, in which the amount of information is rich, and the memory is effective.

Experiment 4 asked the question of how similar are the objects recovered by the memory retrieval operation to the cue or key used as the retrieval descriptor. The results show that high similarity is achieved for very low and moderate levels of entropy. In the basic case, when the entropy is zero, the retrieved object is the same as the cue, and memory recognition and memory retrieval are not distinguished. This corresponds to *Ramdom Access Memories* (RAM) of standard digital computers, where the content of a RAM register is “copied” but not really extracted or recovered in a memory read operation. Natural memories are constructive, in the sense that the memory retrieve operation renders a genuine novel object. This is the reason to define the $\beta$ operator using a random distribution. Whenever the cue is accepted the retrieval operation selects an object whose representation is within the relation’s constituent functions. The retrieved object may or may not have been registered explicitly, but it is the product of a construction operation always. The similarity experiment showed that high resemblance between the cue and the recovered object is achieved when the entropy has low and moderate values using operational registers. If the entropy is zero, the retrieved object is a “photographic copy” of the stored object. The entropy range also suggests that retrieval goes from “photographic” to “recovered objects” to “imaged objects” to noise. Once again, operational memories have an entropy range in which the entropy is not too low and not too high. This pattern seems to be very general and is referred to as *The Entropy Trade-off*^23^.

The constructive nature of the retrieval operation opposes the reproductive property of the autoencoder. In practice the decoder computes only an approximation of the inverse function computed by the encoder, and the input and output shapes are slightly different when the autoencoder is used without recurring to the memory, as can be seen contrasting the first and second row in the digit’s figures above. This effect is due to the associations made by the neural networks, but should not be confused with the constructive nature of the memory retrieval operation that produces the digits in remaining rows at different entropy levels. The associations result from zero entropy computations, but constructions are computations with entropy larger than zero. The difference between the operation of the autoencoder and the memory retrieval operation illustrates the old opposition between associationism versus constructivism in learning theories—e.g., classical behaviorism versus Bartlett’s and even Piaget’s constructivism.

Experiment 5 addressed recognising, retrieving, and rendering information on the basis of incomplete information. This functionality has been one important motivation for the study of associative memory since Bartlett’s work on remembering^2^, and a practical motivation for developing artificial associative memories such as the original Hopfield model. The use of poor cues or incomplete information is an strong test for the system and highlights the effect of the entropy level for the recognition and retrieval operations: precision is high but recall is low at low levels of entropy; at these levels it is difficult to accept the cues and there may be false negatives, but when they are accepted they are recognized correctly. This relation reverses according to the increase of the entropy: There is a moderate range of entropy in which recall and precision are both satisfactory as there are few false negatives and few false positives; however, when the entropy is too high most cues may be accepted, but quite a few interpretations may be wrong.

This experiment highlights as well the main conceptual differences between the present proposal and the associative memories developed within the neural networks paradigm, as follows: (i) while the present model generalize over the stored patterns and allows for imagination, the latter stores the patterns that have been explicitly input, resembling in this respect Random Access Memories of standard digital computers; (ii) while memory retrieval in the present model is a constructive operation rendering a novel object, the network model searches for a previously stored pattern that matches the cue; (iii) while our memory model rejects patterns that are not contained in the memory directly and efficiently, the network models search extensively for a pattern matching the cue; hence memory retrieval either provides always a pattern, which may be a wrong answer, or fails, implementing a form of negation by failure, which is not a true negation, assuming implicitly the closed world assumption; in addition, the prediction in this latter case is that rejecting a pattern would be a costly and time consuming operation, countering common experience and intuition.

This experiment also highlights that the memory retrieval involves a bottom-up operation that produces the abstract characterization of the input and a top-down operation that contributes top-down to the interpretation. This phenomenon is highlighted by the cases in which the output of the analysis network is fed into the synthesis directly—a zero entropy process—but the cue is too poor and the object cannot be reproduced; but can be reconstructed by the memory retrieval operation at an adequate level of entropy. Indeed, the present architecture has a Bayesian orientation although without using explicit probabilities. In this analogy the analysis network computes a *likelihood*; the information in the memory plays the role of a *prior*; and the retrieval operation involves a trade-off between both sources of information for rendering the best object that is consistent with the external evidence—i.e., the cue—and the previous experience stored in memory. The synthesis network can also be thought of as playing the role of likelihood and the rendered object is again the best compromise between the memory and the generation system. This perspective illustrates the impact on memory in perceptual interpretation and in rendering the specification of the output: while the declarative memory allows for the use of previous experience, which is learnt, cognitive architectures lacking the memory rely only in perceptual and motor abilities, which are trained.

The study of memory mechanisms for storing the representations of individual objects is central to cognition and computer applications, such as information systems and robotics. The sensed data may be presented to cognition in a natural format in which spatial and temporal information may be accessed directly, but may be stored as a highly abstract modality-independent representation. Such representations may be retrieved directly by perception in the production of interpretations, by thought in decision making and planning, and by the motricity or a motor module when motor abilities are deployed. Associative memory mechanisms should support long term memory, both episodic and semantic, and may be accessed on demand by working memory. Such devices may be used in the construction of episodic memories and composite concepts that may be stored in associative memory registers themselves, or in higher level structures that rely on basic memory units. Associative memory models may be essential for the construction of lexicons, encyclopedic memories, and modality-specific memories, such as faces, prototypical shapes or voices, both in cognitive studies and applications. The present investigation addressed the design and construction of the full associative memory system for a simple domain, and the current result can be seen as a proof of concept. We left the investigation of larger and more realistic domains for further work.
