# Author Correction: An entropic associative memory

**DOI:** 10.1038/s41598-021-96664-2

**Published:** 2021-08-24

**Authors:** Luis A. Pineda, Gibrán Fuentes, Rafael Morales

**Affiliations:** 1 Universidad Nacional Autónoma de México, IIMAS, 04510 Mexico City, Mexico; 2 Universidad de Guadalajara, SUV, 44130 Guadalajara, Mexico

Correction to: *Scientific Reports* 10.1038/s41598-021-86270-7, published online 25 March 2021.

The original version of this Article contained an error in Figure 3 and 4, where the results of the experiments were incorrect due to a programming bug. The original Figures 3 and 4 and accompanying legends appear below.

**Figure 3:**
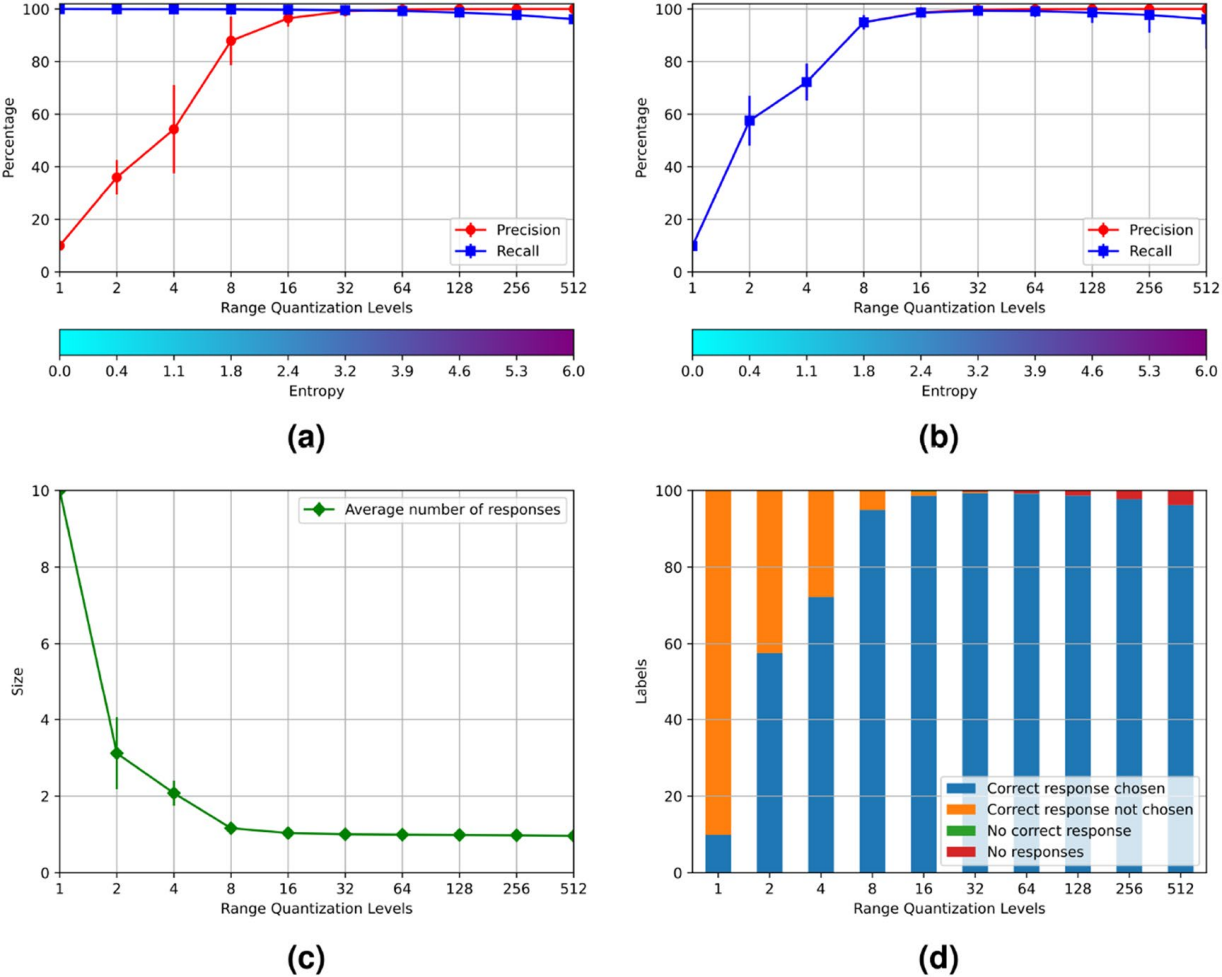
Results of experiment 1.

**Figure 4:**
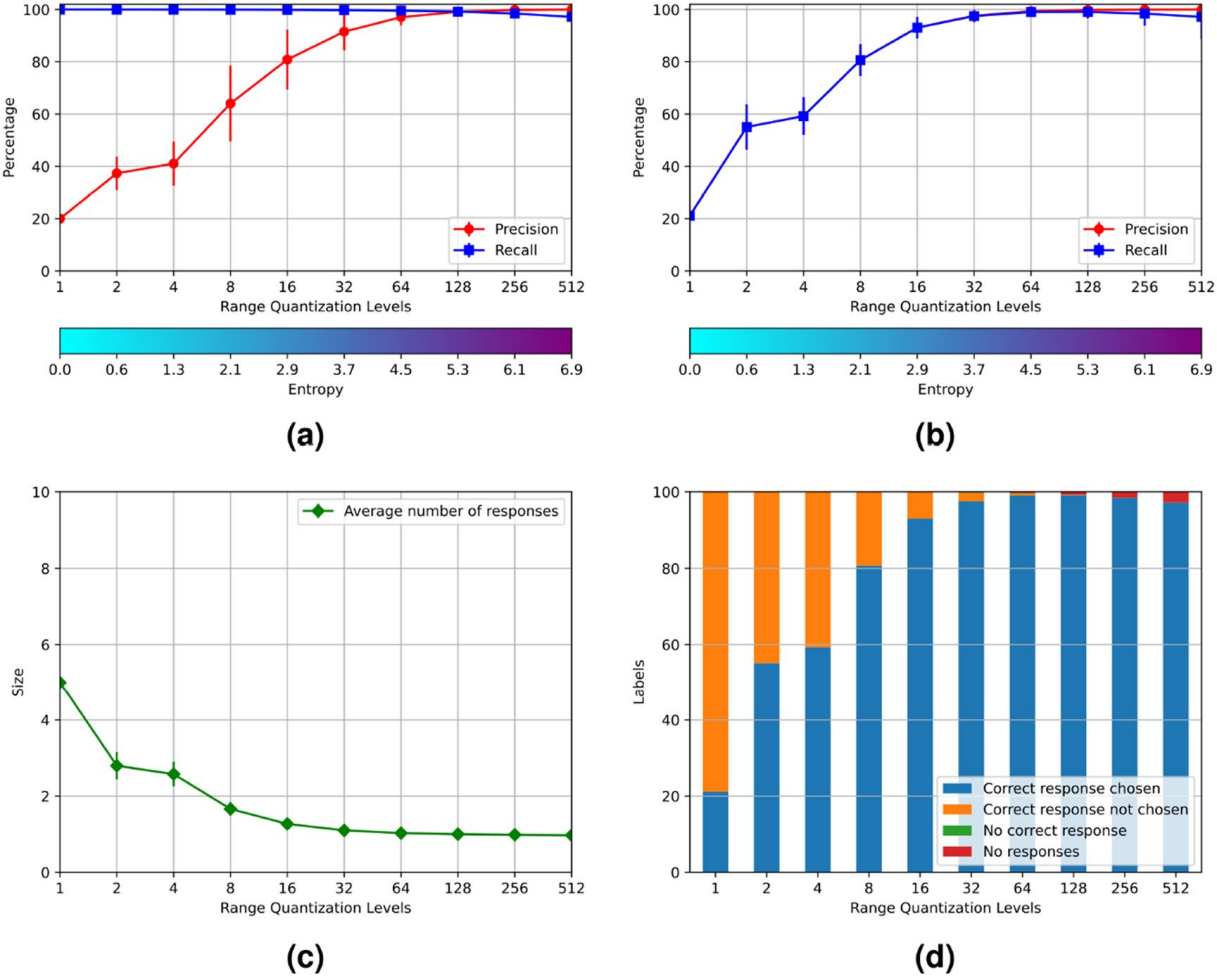
Results of experiment 2.

In addition, in the section A visual memory for hand written digits, under the subheading ‘Experiment 1’,

“The recall, on its part, remains very high until the granularity of the table is too fine and it starts to decrease slightly.”

now reads:

"The recall, on its part, remains high until the granularity of the table is too fine and it starts to decrease."

The original Article has been corrected.

